# Radar nonlinear multi-target tracking method with parallel PHD filter

**DOI:** 10.1038/s41598-024-56065-7

**Published:** 2024-03-04

**Authors:** Jin Tao, Defu Jiang, Jialin Yang, Yan Han, Song Wang, Xingchen Lu

**Affiliations:** 1 School of Computer and Information, Hohai University, Nanjing, 210098 China; 2 Laboratory of Array and Information Processing, Hohai University, Nanjing, 210098 China

**Keywords:** Engineering, Mathematics and computing

## Abstract

Since probability hypothesis density (PHD) filters do not need explicit data association, they have recently been widely used in radar multi-target tracking (MTT). However, in existing PHD filters, sampling times are generally considered the same for all targets. Due to the limitation of antenna beam width in radar applications, the same sampling time for all targets will lead to a mismatch between the predicted data and measurement data, reducing the accuracy of radar MTT. In order to eliminate the estimation error with less computational cost, a radar nonlinear multi-target tracking method with a parallel PHD filter is proposed in this article. The measurement area is divided into several subspaces according to the beam width of the radar antenna, and the PHD of all subspaces is calculated in parallel. Then, multi-feature information in radar echo assists tracking and improves real-time performance. Experimental results in various scenarios illustrate that the proposed method can eliminate the estimation errors introduced by sampling time diversity at the cost of less computation cost, especially in cluttered environments.

## Introduction

Radar MTT plays a vital role in a radar system to estimate multi-target states from radar measurement data in real-time^1–3^. Traditional radar MTT algorithms employ data association techniques, for instance, probabilistic multiple hypothesis tracking (PMHT)^4^, joint probabilistic data association (JPDA)^5^, and multiple hypothesis tracking (MHT)^6^. The time cost increases exponentially when the high clutter rate or the measurement data is extensive. Recently, PHD filters based on random finite sets (RFS) have been widely used in MTT because they do not need explicit data association.

Many approximations of multi-target filters under the Bayes framework have been proposed, such as multi-target multi-Bernoulli filter (MeMBer)^7^, cardinalized PHD filter (CPHD)^8^, Sequential Monte Carlo PHD filter (SMC-PHD)^9–12^, and Gaussian mixture PHD filter (GM-PHD)^13–16^. These filters have been widely used in the field of MTT. To further improve the estimation performance and provide target trajectory information, a labeled multi-Bernoulli (LMB) filter^17^ is proposed, which uses label information to identify different targets. Subsequently, a generalized labeled multi-Bernoulli (GLMB) filter^18,19^ and various improved labeled RFS-based filters^20–23^ have been further developed. However, these methods do not consider the diversity of sampling time in practical radar applications. These methods generally assume the same sampling time for all targets. This assumption will lead to estimation error in the practical radar applications, thus reducing the accuracy of multi-target tracking.

In the radar field, beam scanning technology can make the radar more accurately detect the target's position information. Compared with wide beam technology, narrow beam scanning can achieve accurate positioning, higher detection and signal reception accuracy, longer transmission distance, and better transmission signal quality. However, the narrow beam scanning also has a specific limitation: the beam width is limited. Due to the limitation of antenna beam width, targets located in different spatial locations will be detected by different beams, resulting in the diversity of sampling time in the same scanning period. Therefore, using the same sampling time for all targets will lead to a mismatch between the predicted data and measurement data, reducing the accuracy of radar MTT. To address this issue, a time-matching filter based on RFS in^24^ and a time-matching PHD filter for extended targets in^25^ are proposed for MTT, which divides the sampling time into several parts on average. However, these methods eliminate the estimation errors caused by sampling time diversity at the expense of a higher time cost. Especially in cluttered environments, the time cost is higher, and the real-time performance is seriously degraded. Therefore, this paper proposes a radar nonlinear MTT method with a parallel PHD filter. The measurement space is divided into several subspaces according to the beam width of the radar antenna, and the PHD of all subspaces is calculated in parallel. Meanwhile, in the PHD update step, Doppler and signal-to-noise ratio (SNR) contained in radar echo are considered as features to eliminate clutter and distinguish different targets, further reducing the computational cost. In the application of radar, because different targets have different Doppler frequencies, and the SNR is related to the radar cross section (RCS), range, and speed of the target, which can distinguish the target and clutter, this paper only selects the Doppler frequency and SNR as the feature information. Of course, it is also possible to select one or several other feature information in the radar echo according to the actual situation to distinguish the target and clutter. Compared with the conventional time matching scheme, the proposed method realizes parallel PHD filtering by dividing measurement space to eliminate the estimation error caused by sampling time mismatch. However, the conventional time-matching scheme will increase the calculation cost when the clutter density is high. In contrast, the proposed method uses multi-feature information of the radar echo to eliminate the clutter in the update step and reduce the calculation cost. Simulations indicate that the proposed method eliminates the estimation errors introduced by sampling time diversity with less computation cost and performs well in real-time in cluttered environments.

The following is a summary of the article's main contributions:

First, we proposed a parallel PHD filter for radar nonlinear MTT, which can enhance real-time and radar MTT performance, especially in cluttered environments.

Second, according to the beam width of the radar antenna, the measurement space is divided into several subspaces to eliminate the estimation errors introduced by sampling time diversity, and the PHD of all subspaces is calculated in parallel.

Third, in the PHD update step, Doppler and signal-to-noise ratio (SNR) contained in radar echo are considered as features to assist multi-target tracking; these features can eliminate clutter and differentiate targets from each other, further reducing the computational cost.

## Background

### The problem description of multi-target tracking in radar applications

In the practical application of radar, because of the limitation of antenna beam width, targets located in different spatial locations will be detected by different beams, resulting in sampling time diversity for different targets within the same scanning period.

**Figure 1:**
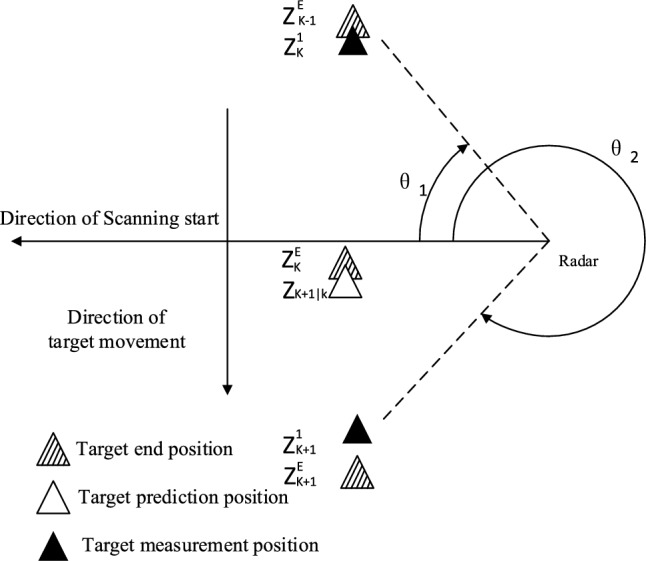
Surveillance area of the mechanical scanning radar.

To illustrate this problem further, Fig. 1 shows the surveillance area of the mechanical scanning radar; among them, the radar adopts the clockwise scanning mode, and the scanning speed is constant. In this scenario, there is a target moving at a constant speed. The target position is ${\varvec{Z}}_{k - 1}^{E}$ at the end of the scan $k - 1$. It will move from ${\varvec{Z}}_{k - 1}^{E}$ to ${\varvec{Z}}_{k}^{E}$ during scan $k$, and move from ${\varvec{Z}}_{k}^{E}$ to ${\varvec{Z}}_{k + 1}^{E}$ during scan $k + 1$. Figure 1 also shows that the scanned positions of the target are respectively nearby ${\varvec{Z}}_{k}^{1}$, ${\varvec{Z}}_{k}^{E}$, and ${\varvec{Z}}_{k + 1}^{1}$. Assuming that the target has been stably tracked before the scan $k$, the target’s predicted position by the filter will appear near ${\varvec{Z}}_{k}^{1}$ in the scan $k$. Since ${\varvec{Z}}_{k}^{1}$ is close to the target’s predicted position, ${\varvec{Z}}_{k}^{1}$ will be treated as the target-generated measurement, which is used to update the prediction priors. Similarly, measurement ${\varvec{Z}}_{k}^{E}$ generated during scan $k$ will be treated as clutter and does not contribute to the posterior calculation of the target. In the scan $k + 1$, the target will move to ${\varvec{Z}}_{k + 1}^{E}$ and be detected at ${\varvec{Z}}_{k + 1}^{1}$. According to the calculation result of the scan $k$, the target predicted position by the filter will be close to ${\varvec{Z}}_{k + 1|k}$. If the position is too different from the actual value, the filter may regard ${\varvec{Z}}_{k + 1|k}$ as clutter and discard it. Meanwhile, the missed detection probability is used to compensate for the information loss due to the lost measurement, resulting in the divergence of the filter.

Because the standard PHD filter does not consider the time information of the multi-target states, it is generally assumed that the measurements of targets are generated simultaneously, so the multi-target states cannot be accurately estimated.

### RFS-based PHD filters

The PHD filter based on RFS is an approximate Bayesian filter that propagates the first moment rather than the entire multi-target posterior^10^. Among them, PHD is defined as an intensity function, expressed as^26^:

1 $$D({\varvec{x}}) = \int {\pi ({\varvec{x}} \cup W)\delta W = } \int_{{{\varvec{x}} \in {\varvec{X}}}} {\pi ({\varvec{X}})\delta {\varvec{X}}}$$

The multi-target state at time $k$ is naturally denoted as finite sets in RFS-based PHD filters:

2 $${\varvec{X}}_{k} = \{ {\varvec{x}}_{k}^{1} ,{\varvec{x}}_{k}^{2} ,...,{\varvec{x}}_{k}^{M(k)} \} \in F({\mathbf{\mathbb{X}}})$$

The multi-target measurement at time $k$ is denoted by:

3 $${\varvec{Z}}_{k} = \{ {\varvec{z}}_{k}^{1} ,{\varvec{z}}_{k}^{2} ,...,{\varvec{z}}_{k}^{N(k)} \} \in F({\mathbf{\mathbb{Z}}})$$

where $M(k)$ and $N(k)$ are target number and measurement number respectively, $F({\mathbf{\mathbb{X}}})$ and $F({\mathbf{\mathbb{Z}}})$ respectively represent the collection of all finite subsets of the state space ${\mathbf{\mathbb{X}}}$ and ${\mathbf{\mathbb{Z}}}$.

The PHD filter propagates the intensity function in time through two steps: prediction and update^10^. Let $D_{k|k} ({\varvec{x}})$ denote the intensity function, the predictor operator $D_{k|k - 1} ({\varvec{x}})$ is

4 $$D_{k|k - 1} ({\varvec{x}}) = b_{k|k - 1} ({\varvec{x}}) + \int {F_{k|k - 1} } ({\varvec{x}}|\user2{x^{\prime}})D_{k - 1} (\user2{x^{\prime}})d\user2{x^{\prime}}$$

5 $$F_{k|k - 1} ({\varvec{x}}|\user2{x^{\prime}}) = b_{k|k - 1} ({\varvec{x}}|\user2{x^{\prime}}) + P_{S,k} (\user2{x^{\prime}})f_{k|k - 1} ({\varvec{x}}|\user2{x^{\prime}})$$

with $b_{k|k - 1} ({\varvec{x}})$ designating the PHD of targets for spontaneous birth, $b_{k|k - 1} ({\varvec{x}}|\user2{x^{\prime}})$ designating the PHD of targets for spawned, $f_{k|k - 1} ({\varvec{x}}|\user2{x^{\prime}})$ denoting the object transition kernel, and $P_{S,k} (\user2{x^{\prime}})$ denoting the target's survival probability.

The update operator is

6 $$D_{k|k} ({\varvec{x}}) = L_{{{\varvec{Z}}_{k} }} ({\varvec{x}})D_{k|k - 1} ({\varvec{x}})$$

7 $$L_{{{\varvec{Z}}_{k} }} ({\varvec{x}}) = 1 - P_{D,k} ({\varvec{x}}) + \sum\limits_{{{\varvec{z}} \in {\varvec{Z}}_{k} }} {\frac{{P_{D,k} ({\varvec{x}})g_{{\varvec{z}}} ({\varvec{x}})}}{{\lambda_{k} c_{k} ({\varvec{z}}) + D_{k|k - 1} [P_{D,k} g_{{\varvec{z}}} ]}}}$$

with $g_{{\varvec{z}}} ({\varvec{x}})$ denoting the likelihood of individual targets, $P_{D,k} ({\varvec{x}})$ denoting the target's detection probability, $\lambda_{k} c_{k} ({\varvec{z}})$ denoting the PHD of clutter, and $D_{k|k - 1} [h] = \int {h({\varvec{x}})D_{k|k - 1} ({\varvec{x}})d{\varvec{x}}}$.

## The proposed method

### The parallel probability hypothesis density filter

As shown in Eqs. (4) and (6), the PHD filter does not consider the sampling time diversity of different targets, leading to the estimation error between prediction and measurement. Assuming that the radar needs $N_{S}$ beams to scan the whole measurement area, the measurement area is divided into $N_{S}$ independent subspaces. Since the scanning time of each subspace is very short, the sampling time diversity of each subspace can be ignored. Therefore, the multi-target state can be expressed as follows:

8 $${\varvec{X}} = \{ {\varvec{X}}^{{S_{1} }} , \ldots {\varvec{X}}^{{S_{i} }} , \ldots {\varvec{X}}^{{S_{{N_{S} }} }} \} \in F({\mathbf{\mathbb{X}}}^{{S_{1} }} ) \times \cdot \cdot \cdot \times F({\mathbf{\mathbb{X}}}^{{S_{{N_{S} }} }} )$$

with ${\varvec{X}}^{{S_{i} }}$ denoting the multi-target state whose projection on the measurement space falls on subspace $i$, and ${\mathbf{\mathbb{X}}}^{{S_{i} }}$ denoting the state subspace projected onto subspace $i$ in the state space ${\mathbf{\mathbb{X}}}$. According to the above state model, the PHD can be expressed as follows:

9 $$\begin{aligned} D({\varvec{x}}) & = \int_{{{\varvec{X}} \mathrel\backepsilon {\varvec{x}}}} {\pi ({\varvec{X}})\delta {\varvec{X}}} \\ & { = }\int_{{(({\varvec{X}} - {\varvec{X}}^{{S_{i} }} ){ \uplus }{\varvec{X}}^{{S_{i} }} ) \mathrel\backepsilon {\varvec{x}}}} {\pi ((} {\varvec{X}} - {\varvec{X}}^{{S_{i} }} ){ \uplus }{\varvec{X}}^{{S_{i} }} )\delta (({\varvec{X}} - {\varvec{X}}^{{S_{i} }} ){ \uplus }{\varvec{X}}^{{S_{i} }} ) \\ & { = }\sum\limits_{i = 1}^{{N_{S} }} {\int_{{{\varvec{X}}^{{S_{i} }} \mathrel\backepsilon {\varvec{x}}}} {\int_{{{\mathbf{\mathbb{X}}} - {\mathbf{\mathbb{X}}}^{{S_{i} }} {\mathbf{\ominus }}}} {\pi (({\varvec{X}} - {\varvec{X}}^{{S_{i} }} ){ \uplus }{\varvec{X}}^{{S_{i} }} )\delta } } } ({\varvec{X}} - {\varvec{X}}^{{S_{i} }} )\delta {\varvec{X}}^{{S_{i} }} \\ \end{aligned}$$

where symbol ${ \uplus }$ stands for the disjoint union, for arbitrary sets $A$ and $B$, $C = A{ \uplus }B$ is equivalent to $C = A \cup B$ and $A \cap B = \emptyset$.

According to the independence of targets between subspaces, Eq. (9) can be further rewritten as the sum of PHD in different subspaces. An indicator function that accepts any arguments, including vectors, sets, etc., is denoted by

10 $$1_{Y} (X) = \left\{ {\begin{array}{*{20}l} {1,} \hfill & {{\text{ if }}X \subseteq Y,{\text{or }}X \in Y} \hfill \\ {0,} \hfill & {{\text{otherwise}}} \hfill \\ \end{array} } \right.$$

Then the PHD based on parallel processing is expressed as follows:

11 $$\begin{aligned} D({\varvec{x}}) & = \sum\limits_{i = 1}^{{N_{S} }} {\int_{{{\varvec{X}}^{{S_{i} }} \mathrel\backepsilon {\varvec{x}}}} {\pi (} } {\varvec{X}}^{{S_{i} }} )\int_{{{\mathbf{{\mathbb{X}} }} - {\mathbf{\mathbb{X}}}^{{S_{i} }} }} {\pi ({\varvec{X}} - } {\varvec{X}}^{{S_{i} }} )\delta ({\varvec{X}}^{{S_{i} }} )\delta {\varvec{X}}^{{S_{i} }} \\ \, & { = }\sum\limits_{i = 1}^{{N_{S} }} {\int_{{{\varvec{X}}^{{S_{i} }} \mathrel\backepsilon {\varvec{x}}}} {\pi (} } {\varvec{X}}^{{S_{i} }} )\delta {\varvec{X}}^{{S_{i} }} \\ \, & { = }\sum\limits_{i = 1}^{{N_{S} }} {1_{{{\mathbf{\mathbb{X}}}^{{S_{i} }} }} } ({\varvec{x}})D^{{S_{i} }} ({\varvec{x}}) \\ \end{aligned}$$

with $D^{{S_{i} }} ({\varvec{x}})$ denoting the PHD of all targets in subspace $i$. Let $D_{k - 1} ({\varvec{x}})$ denote the posterior PHD obtained at the $k - 1$ scan cycle. According to Eq. (11), the predicted PHD of the scan $k$ is expressed as:

12 $$\begin{aligned} D_{k|k - 1} ({\varvec{x}}) & = \sum\limits_{i = 1}^{{N_{S} }} {1_{{{\mathbf{\mathbb{X}}}^{{S_{i} }} }} (} {\varvec{x}})D_{k|k - 1}^{{S_{i} }} ({\varvec{x}}) \\ & { = }\sum\limits_{i = 1}^{{N_{S} }} {1_{{{\mathbf{\mathbb{X}}}^{{S_{i} }} }} (} {\varvec{x}})\left( {b_{k|k - 1}^{{S_{i} }} ({\varvec{x}}) + \int {F_{k|k - 1} } ({\varvec{x}}|\user2{x^{\prime}})D_{k - 1} (\user2{x^{\prime}})d\user2{x^{\prime}}} \right) \\ & { = }\sum\limits_{i = 1}^{{N_{S} }} {1_{{{\mathbf{\mathbb{X}}}^{{S_{i} }} }} (} {\varvec{x}})\left( {b_{k|k - 1}^{{S_{i} }} ({\varvec{x}}) + \int {F_{k|k - 1} } ({\varvec{x}}|\user2{x^{\prime}})\sum\limits_{j = 1}^{{N_{S} }} {1_{{{\mathbf{\mathbb{X}}}^{{S_{j} }} }} } (\user2{x^{\prime}})D_{k - 1}^{{S_{j} }} (\user2{x^{\prime}})d\user2{x^{\prime}}} \right) \\ & { = }\sum\limits_{i = 1}^{{N_{S} }} {1_{{{\mathbf{\mathbb{X}}}^{{S_{i} }} }} (} {\varvec{x}})\left( {b_{k|k - 1}^{{S_{i} }} ({\varvec{x}}) + \sum\limits_{j = 1}^{{N_{S} }} {\int_{{{\mathbf{\mathbb{X}}}^{{S_{j} }} }} {F_{k|k - 1} ({\varvec{x}}|\user2{x^{\prime}})} } D_{k - 1}^{{S_{j} }} (\user2{x^{\prime}})d\user2{x^{\prime}}} \right) \\ & { = }\sum\limits_{i = 1}^{{N_{S} }} {1_{{{\mathbf{\mathbb{X}}}^{{S_{i} }} }} (} {\varvec{x}})\left( {b_{k|k - 1}^{{S_{i} }} ({\varvec{x}}) + \sum\limits_{j = 1}^{{N_{S} }} {D_{k|k - 1}^{{S_{i} |S_{j} }} } ({\varvec{x}})} \right) \\ \end{aligned}$$

with $b_{k|k - 1}^{{S_{i} }} ({\varvec{x}})$ denoting the PHD of all birth targets in subspace $i$. The PHD of all birth targets in the surveillance area is

13 $$b_{k|k - 1} ({\varvec{x}}) = \sum\limits_{i = 1}^{{N_{S} }} {1_{{{\mathbf{\mathbb{X}}}^{{S_{i} }} }} } ({\varvec{x}})b_{k|k - 1}^{{S_{i} }} ({\varvec{x}})$$

The measurement model can be expressed as follows, which is analogous to the multi-target state model described in Eq. (8):

14 $${\varvec{Z}}_{k} = \{ {\varvec{Z}}_{k}^{{S_{1} }} ,...{\varvec{Z}}_{k}^{{S_{i} }} ,...{\varvec{Z}}_{k}^{{S_{{N_{S} }} }} \} \in F({\mathbf{{\mathbb{Z}}\ }}^{{S_{1} }} ) \times \cdot \cdot \cdot \times F({\mathbf{\mathbb{Z}}}^{{S_{{N_{S} }} }} )$$

where ${\varvec{Z}}_{k}^{{S_{i} }}$ is the measurement set from subspace $i$.

The following is the written form of the PHD update operator:

15 $$\begin{aligned} D_{k|k} ({\varvec{x}}) & = (1 - P_{D,k} ({\varvec{x}}))D_{k|k - 1} ({\varvec{x}}) + \sum\limits_{{{\varvec{z}} \in {\varvec{Z}}_{k} }} {\frac{{P_{D,k} ({\varvec{x}})g_{{\varvec{z}}} ({\varvec{x}})D_{k|k - 1} ({\varvec{x}})}}{{\lambda_{k} c_{k} ({\varvec{z}}) + D_{k|k - 1} [P_{D,k} g_{{\varvec{z}}} ]}}} \\ & = (1 - P_{D,k} ({\varvec{x}}))D_{k|k - 1} ({\varvec{x}}) + \sum\limits_{i = 1}^{{N_{S} }} {\sum\limits_{{{\varvec{z}} \in {\varvec{Z}}_{k}^{{S_{i} }} }} {\frac{{P_{D,k} ({\varvec{x}})g_{{\varvec{z}}} ({\varvec{x}})D_{k|k - 1} ({\varvec{x}})}}{{\lambda_{k} c_{k} ({\varvec{z}}) + D_{k|k - 1} [P_{D,k} g_{{\varvec{z}}} ]}}} } \\ \end{aligned}$$

with $g_{{\varvec{z}}} ({\varvec{x}})$ denoting the likelihood function of the measurement ${\varvec{z}}$. According to the independence of targets between subspaces, for arbitrary state ${\varvec{x}}$ and measurement ${\varvec{z}}$, if ${\varvec{x}} \notin {\mathbf{{\mathbb{X}}\ominus }}^{{S_{i} }}$ and ${\varvec{z}} \in {\varvec{Z}}_{k}^{{S_{i} }}$, we have $g_{{\varvec{z}}} ({\varvec{x}}) = 0$. Therefore, Eq. (15) is further rewritten as:

16 $$\begin{aligned} D_{k|k} ({\varvec{x}}) & = \sum\limits_{i = 1}^{{N_{S} }} {1_{{{\mathbf{\mathbb{X}}}^{{S_{i} }} }} } ({\varvec{x}})(1 - P_{D,k} ({\varvec{x}}))D_{k|k - 1}^{{S_{i} }} ({\varvec{x}}) + \sum\limits_{i = 1}^{{N_{S} }} {\sum\limits_{{{\varvec{z}} \in {\varvec{Z}}_{k}^{{S_{i} }} }} {\frac{{P_{D,k} ({\varvec{x}})1_{{{\mathbf{\mathbb{X}}}^{{S_{i} }} }} ({\varvec{x}})g_{{\varvec{z}}} ({\varvec{x}})D_{k|k - 1}^{{S_{i} }} ({\varvec{x}})}}{{\lambda_{k} c_{k} ({\varvec{z}}) + D_{k|k - 1}^{{S_{i} }} [P_{D,k} g_{{\varvec{z}}} ]}}} } \\ & = \sum\limits_{i = 1}^{{N_{S} }} {1_{{{\mathbf{\mathbb{X}}}^{{S_{i} }} }} } ({\varvec{x}})D_{k|k - 1}^{{S_{i} }} ({\varvec{x}}) \times \left( {(1 - P_{D,k} ({\varvec{x}})) + \sum\limits_{{{\varvec{z}} \in {\varvec{Z}}_{k}^{{S_{i} }} }} {\frac{{P_{D,k} ({\varvec{x}})g_{{\varvec{z}}} ({\varvec{x}})}}{{\lambda_{k} c_{k} ({\varvec{z}}) + D_{k|k - 1}^{{S_{i} }} [P_{D,k} g_{{\varvec{z}}} ]}}} } \right) \\ & = \sum\limits_{i = 1}^{{N_{S} }} {1_{{{\mathbf{\mathbb{X}}}^{{S_{i} }} }} } ({\varvec{x}})D_{k|k}^{{S_{i} }} ({\varvec{x}}) \\ \end{aligned}$$

with $D_{k|k - 1}^{{S_{i} }} [P_{D,k} g_{{\varvec{z}}} ]$ denoting the single target state subspace ${\mathbf{\mathbb{X}}}^{{S_{i} }}$ projected onto subspace $i$, specifically as follows:

17 $$D_{k|k - 1}^{{S_{i} }} [P_{D,k} g_{{\varvec{z}}} ] = \int_{{{\mathbf{\mathbb{X}}}^{{S_{i} }} }} {P_{D,k} ({\varvec{x}})g_{{\varvec{z}}} } ({\varvec{x}})D_{k|k - 1}^{{S_{i} }} ({\varvec{x}})d{\varvec{x}}$$

### The proposed method's specific steps

**Figure 2:**
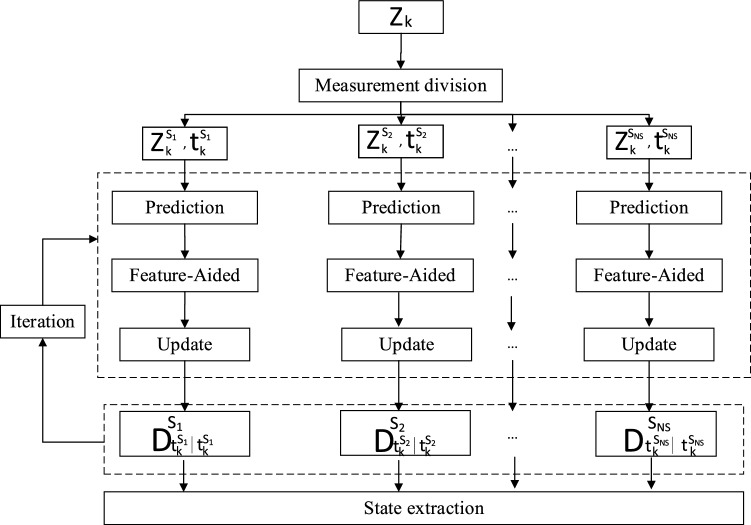
The proposed method's specific steps.

In Eq. (16), PHD is denoted by the sum of the PHD components of different subspaces. Compared with the product form, this form of accumulation has better parallel processing ability, so it is suitable for parallel filtering scenarios in radar applications. This paper will use this framework to propose a radar nonlinear multi-target tracking method based on a parallel PHD filter that can eliminate the estimation errors introduced by sampling time diversity with less computation cost. The specific steps of the proposed method are shown in Fig. 2.

### Measurement division

**T**he measurement area is divided into $N_{S}$ subspaces; targets in the same subspace can be regarded as having the same sampling time. $t_{k}^{{S_{i} }}$ represents the sampling time of subspace $i$, the multi-target state is expressed as follows:

18 $${\varvec{X}}_{k} = \{ ({\varvec{X}}_{k}^{{^{{S_{1} }} }} ,t_{k}^{{^{{S_{1} }} }} ), \ldots ,({\varvec{X}}_{k}^{{^{{S_{{N_{S} }} }} }} ,t_{k}^{{S_{{N_{S} }} }} )\} \in (F({\mathbf{\mathbb{X}}}^{{S_{1} }} ) \times {\mathbf{\mathbb{T}}}^{{S_{1} }} ) \times \cdot \cdot \cdot \times (F({\mathbf{\mathbb{X}}}^{{S_{{N_{S} }} }} ) \times {\mathbf{\mathbb{T}}}^{{S_{{N_{S} }} }} )$$

with ${\mathbf{\mathbb{T}}}^{{S_{i} }}$ denoting the sampling time space of the target in subspace $i$, and the multi-target measurement is expressed as follows:

19 $${\varvec{Z}}_{k} = \{ ({\varvec{Z}}_{k}^{{S_{1} }} ,t_{k}^{{S_{1} }} ), \ldots ,({\varvec{Z}}_{k}^{{S_{{N_{S} }} }} ,t_{k}^{{S_{{N_{S} }} }} )\} \in (F({\mathbf{{\mathbb{Z}}\ }}^{{S_{1} }} ) \times {\mathbf{\mathbb{T}}}^{{S_{1} }} ) \times \cdot \cdot \cdot \times (F({\mathbf{\mathbb{Z}}}^{{S_{{N_{S} }} }} ) \times {\mathbf{\mathbb{T}}}^{{S_{{N_{S} }} }} )$$

### Prediction

Each subspace takes the same sampling time, similar to the division of PHD in Eq. (11), then the PHD is expressed as:

20 $$D_{k|k} ({\varvec{x}}) = \sum\limits_{i = 1}^{{N_{S} }} {1_{{{\mathbf{\mathbb{X}}}^{{S_{i} }} }} } ({\varvec{x}})D_{{t_{k}^{{S_{i} }} |t_{k}^{{S_{i} }} }}^{{S_{i} }} ({\varvec{x}})$$

with $D_{{t_{k}^{{S_{i} }} |t_{k}^{{S_{i} }} }}^{{S_{i} }} ({\varvec{x}})$ denoting the PHD of subspace $i$ at time $t_{k}^{{S_{i} }}$. Let $D_{k - 1|k - 1} ({\varvec{x}})$ denote the posterior PHD of the scan $k - 1$. According to Eq. (20), the predicted PHD of the $k$ scan cycle can be defined by:

21 $$\begin{aligned} D_{k|k - 1} ({\varvec{x}}) & = \sum\limits_{i = 1}^{{N_{S} }} {1_{{{\mathbf{\mathbb{X}}}^{{S_{i} }} }} } ({\varvec{x}})D_{{t_{k}^{{S_{i} }} |k - 1}}^{{S_{i} }} ({\varvec{x}}) \\ & { = }\sum\limits_{i = 1}^{{N_{S} }} {1_{{{\mathbf{\mathbb{X}}}^{{S_{i} }} }} (} {\varvec{x}})\left( {b_{{t_{k}^{{S_{i} }} |k - 1}}^{{S_{i} }} ({\varvec{x}}) + \int {F_{{t_{k}^{{S_{i} }} |k - 1}} } ({\varvec{x}}|\user2{x^{\prime}})D_{k - 1|k - 1} (\user2{x^{\prime}})d\user2{x^{\prime}}} \right) \\ & { = }\sum\limits_{i = 1}^{{N_{S} }} {1_{{{\mathbf{\mathbb{X}}}^{{S_{i} }} }} (} {\varvec{x}})\left( {b_{{t_{k}^{{S_{i} }} |k - 1}}^{{S_{i} }} ({\varvec{x}}) + \sum\limits_{j = 1}^{{N_{S} }} {\int_{{{\mathbf{\mathbb{X}}}^{{S_{j} }} }} {F_{{t_{k}^{{S_{i} }} |t_{k - 1}^{{S_{j} }} }} ({\varvec{x}}|\user2{x^{\prime}})} } D_{{t_{k - 1}^{{S_{j} }} |t_{k - 1}^{{S_{j} }} }}^{{S_{j} }} (\user2{x^{\prime}})d\user2{x^{\prime}}} \right) \\ & { = }\sum\limits_{i = 1}^{{N_{S} }} {1_{{{\mathbf{\mathbb{X}}}^{{S_{i} }} }} (} {\varvec{x}})\left( {b_{{t_{k}^{{S_{i} }} |k - 1}}^{{S_{i} }} ({\varvec{x}}) + \sum\limits_{j = 1}^{{N_{S} }} {D_{{t_{k}^{{S_{i} }} |t_{k - 1}^{{S_{j} }} }}^{{S_{i} |S_{j} }} } ({\varvec{x}})} \right) \, \\ \end{aligned}$$

with $b_{{t_{k}^{{S_{i} }} |k - 1}}^{{S_{i} }} ({\varvec{x}})$ denoting all birth targets PHD in subspace $i$, $D_{{t_{k}^{{S_{i} }} |t_{k - 1}^{{S_{j} }} }}^{{S_{i} |S_{j} }} ({\varvec{x}})$ denoting the PHD of targets from $t_{k - 1}^{{S_{j} }}$ to $t_{k}^{{S_{i} }}$. Note that $F_{{t_{k}^{{S_{i} }} |t_{k - 1}^{{S_{j} }} }} ({\varvec{x}}|\user2{x^{\prime}})$ can be expressed as:

22 $$F_{{t_{k}^{{S_{i} }} |t_{k - 1}^{{S_{j} }} }} ({\varvec{x}}|\user2{x^{\prime}}) = b_{{t_{k}^{{S_{i} }} |t_{k - 1}^{{S_{j} }} }} ({\varvec{x}}|\user2{x^{\prime}}) + P_{{S,t_{k}^{{S_{i} }} }} (\user2{x^{\prime}})f_{{t_{k}^{{S_{i} }} |t_{k - 1}^{{S_{j} }} }} ({\varvec{x}}|\user2{x^{\prime}})$$

with $b_{{t_{k}^{{S_{i} }} |t_{k - 1}^{{S_{j} }} }} ({\varvec{x}}|\user2{x^{\prime}})$ denoting the PHD of spawned target at time $t_{k}^{{S_{i} }}$, $P_{{S,t_{k}^{{S_{i} }} }} (\user2{x^{\prime}})$ denoting the targets survival probability at time $t_{k}^{{S_{i} }}$, and $f_{{t_{k}^{{S_{i} }} |t_{k - 1}^{{S_{j} }} }} ({\varvec{x}}|\user2{x^{\prime}})$ denoting the transition density of individual targets from $t_{k - 1}^{{S_{j} }}$ to $t_{k}^{{S_{i} }}$.

### Feature-aided

The standard PHD filter uses Eq. (6) to update and iterate repeatedly. In the whole iteration process, $g_{{\varvec{z}}} ({\varvec{x}})$ in Eq. (7) affects the filtering performance, but its calculation only uses the range and bearing information in the measurement. Considering that radar echoes contain abundant feature information in practical applications, such as range, bearing, amplitude, angle, Doppler frequency and SNR. Therefore, in this paper, Doppler and SNR contained in radar echo are considered as features to assist multi-target tracking because these two features of targets and clutter are different, and they are different for various targets. As a result, using these two features can reduce the relatively high computational cost by eliminating much clutter and distinguishing different targets.

In this paper, each target contains feature state ${\varvec{x}}_{{f_{k} }} = [x_{{f_{d} ,k}} ,x_{{P_{SNR} ,k}} ]^{T}$ in addition to kinematic state ${\varvec{x}}_{k} = [x_{k} ,y_{k} ,\dot{x}_{k} ,\dot{y}_{k} ,\ddot{x}_{k} ,\ddot{y}_{k} ]^{T}$, where $x_{{f_{d} ,k}}$ represents the Doppler frequency, $x_{{P_{SNR} ,k}}$ represents the SNR of the target at time $k$, $[\dot{x}_{k} ,\dot{y}_{k} ]^{T}$ and $[x_{k} ,y_{k} ]^{T}$ represent the velocity and the position, respectively, $[\ddot{x}_{k} ,\ddot{y}_{k} ]^{T}$ represents the acceleration. The state of each target is the concatenation of kinematic and feature state, denoted as $\tilde{\user2{x}}_{k} = [{\varvec{x}}_{k} ,{\varvec{x}}_{{f_{k} }} ]^{T}$. Similarly, the corresponding target observation $\tilde{\user2{z}}_{k} = [{\varvec{z}}_{k} ,{\varvec{z}}_{{f_{k} }} ]^{T}$ is the concatenation of kinematic and feature measurement, where kinematic measurement ${\varvec{z}}_{k} = [r,\theta ]^{T} + {\varvec{w}}_{k}$, range $r = \sqrt {x_{k}^{2} + y_{k}^{2} }$, bearing $\theta = \arctan 2(y_{k} ,x_{k} )$ and ${\varvec{w}}_{k}$ is the observation noise; feature measurement ${\varvec{z}}_{{f_{k} }} = [z_{{f_{d} ,k}} ,z_{{P_{SNR} ,k}} ]^{T}$. Consequently, this paper uses feature-likelihood to refine the likelihood function of measurement in Eq. (15), as follows

23 $$g_{{\varvec{z}}} (\tilde{\user2{z}}_{k} |\tilde{\user2{x}}_{k}^{(i)} ) = g_{{\varvec{z}}} ({\varvec{z}}_{k} |{\varvec{x}}_{k}^{(i)} ) \cdot p_{f} ({\varvec{z}}_{{f_{k} }}^{(i)} )$$

24 $$p_{f} ({\varvec{z}}_{{f_{k} }}^{(i)} ) = p_{f} ({\varvec{z}}_{{f_{d,k} }}^{(i)} ) \cdot p_{f} ({\varvec{z}}_{{P_{SNR} ,k}}^{(i)} )$$

with $g_{{\varvec{z}}} ({\varvec{z}}_{k} |{\varvec{x}}_{k}^{(i)} )$ denoting the likelihood function of measurement ${\varvec{z}}_{k}$ generated by single target state ${\varvec{x}}_{k}$ of subspace $i$ at time $t_{k}^{{S_{i} }}$, $p_{f} ({\varvec{z}}_{{f_{k} }}^{(i)} )$ denoting the feature-likelihood of subspace $i$ at time $t_{k}^{{S_{i} }}$. Note that $p_{f} ({\varvec{z}}_{{f_{d,k} }}^{(i)} )$ and $p_{f} ({\varvec{z}}_{{P_{SNR} ,k}}^{(i)} )$ denote the Doppler frequency feature- likelihood and SNR feature-likelihood respectively, as follows:

25 $$p_{f} ({\varvec{z}}_{{f_{d} ,k}}^{(i)} ) = N({\varvec{z}}_{{f_{d} ,k}}^{(i)} ;f_{d,k} ,\sigma_{{f_{d} }}^{2} )$$

26 $$p_{f} ({\varvec{z}}_{{p_{SNR} ,k}}^{(i)} ) = N({\varvec{z}}_{{p_{SNR} ,k}}^{(i)} ;p_{SNR,k} ,\sigma_{{p_{SNR} }}^{2} )$$

where $\sigma_{{f_{d} }}^{2}$ and $\sigma_{{p_{SNR} }}^{2}$ reflect the weight of the Doppler frequency feature- likelihood and SNR feature-likelihood in the calculation of the total likelihood function, in this paper, $\sigma_{{f_{d} }}^{2}$ = $\sigma_{{p_{SNR} }}^{2}$ = 0.85 . As a result, when measurements are closer to the clutter than to the target in probability, the likelihood function declines faster, and the clutter can be greatly reduced. In this way, the comparatively high computational cost is decreased while the real-time performance is enhanced.

### Update

According to Eqs. (16) and (23), the PHD update operator at scan $k$ can be defined by:

27 $$D_{k|k} ({\varvec{x}}) = \sum\limits_{i = 1}^{{N_{S} }} {1_{{{\mathbf{\mathbb{X}}}^{{S_{i} }} }} } ({\varvec{x}})L_{{{\varvec{Z}}_{k}^{{S_{i} }} }} ({\varvec{x}})D_{{t_{k}^{{S_{i} }} |k - 1}}^{{S_{i} }} ({\varvec{x}}) = \sum\limits_{i = 1}^{{N_{S} }} {1_{{{\mathbf{\mathbb{X}}}^{{S_{i} }} }} } ({\varvec{x}})D_{{t_{k}^{{S_{i} }} |t_{k}^{{S_{i} }} }}^{{S_{i} }} ({\varvec{x}})$$

28 $$L_{{{\varvec{Z}}_{k}^{{S_{i} }} }} ({\varvec{x}}) = (1 - P_{{D,t_{k}^{i} }} ({\varvec{x}})) + \sum\limits_{{{\varvec{z}} \in {\varvec{Z}}_{k}^{{S_{i} }} }} {\frac{{P_{{D,t_{k}^{i} }} ({\varvec{x}})g_{{\varvec{z}}} (\tilde{\user2{z}}_{k} |\tilde{\user2{x}}_{k}^{(i)} )}}{{\lambda_{{t_{k}^{{S_{i} }} }} c_{{t_{k}^{{S_{i} }} }} ({\varvec{z}}) + D_{{t_{k}^{{S_{i} }} |k - 1}}^{{S_{i} }} [P_{{D,t_{k}^{i} }} g_{{\varvec{z}}} ]}}}$$

with $P_{{D,t_{k}^{i} }} ({\varvec{x}})$ denoting the detection probability, and $\lambda_{{t_{k}^{{S_{i} }} }} c_{{t_{k}^{{S_{i} }} }} ({\varvec{z}})$ denoting the clutter density at time $t_{k}^{{S_{i} }}$.

Finally, multi-target state estimation is extracted. This method divides the measurement area into several subspaces based on the radar antenna's beam width, and the PHD of all subspaces is calculated in parallel. At the same time, multi-feature information in radar echo is used to assist tracking.

## Simulation and result analysis

Three simulation examples are used to assess the effectiveness of the proposed method. We compare the proposed parallel PHD filter based on feature-aided (FAP-PHD) against the TM-Joint-GLMB filter^24^, TM-PHD filter^25^, and the standard PHD filter through their Gaussian mixture implementation to simulate. The simulations in three different scenarios are shown as follows.

### Simulation scene setting

**Figure 3:**
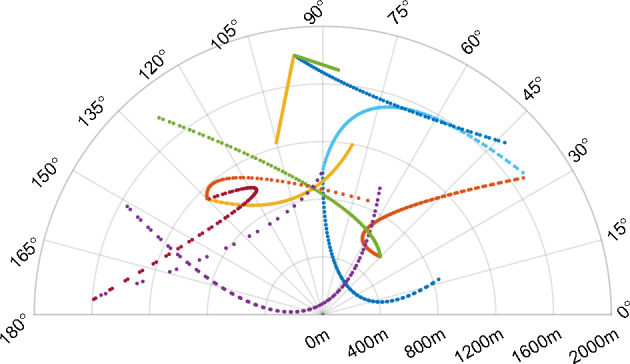
True trajectories of targets.

Scenario 1: A mechanical scanning radar is located at (0 m, 0 m) and scans a semicircle region with the scenario setting range of [0 m, 2000 m] and the angle of [0°, 180°]. The radar uses a bidirectionally continuous scanning mode, i.e., it scans repeatedly from 180° to 0° in a clockwise direction and then from 0° to 180° in an anticlockwise direction. The scanning speed is set to 180°/s. The surveillance region contains twelve targets. Figure 3 shows the true target trajectories with common scenes such as target birth, target death, target turning, and trajectory crossover, and Table 1 shows the initial states and lifetimes of targets. The semicircle region is equally divided into twenty subspaces; each subspace covers 9°. Radar range resolution is 10 m, and radar azimuth resolution is 1°; other scenarios are the same. In the surveillance region, clutter is evenly distributed, and the average clutter rate is $\lambda$ = 50 points for each scan. In this scenario, the detection probability for each target is $P_{D,k}$ = 0.98, and the survival probability for each target is $P_{S,k}$ = 0.99.

**Table 1:** The lifetimes and initial states of targets.

Target Index	Initial states (m, m/s, m/s^2^, m, m/s, m/s^2^)	Lifetime (s)
1	[0, 0, 0.33, 1000, − 37, 0.75]	(1, 70)
2	[400, − 10, 0.4, 400, 5, 0.01]	(1, 100)
3	[− 800, 20, − 0.16, 800, − 5, 0.3]	(1, 70)
4	[400, − 7, − 0.37, 900, − 42, 1]	(20, 100)
5	[400, − 1.4, − 0.44, 400, 10, 0.05]	(20, 100)
6	[0, 5.5, 0.29, 1000, 22, − 0.55]	(20, 100)
7	[− 800, 32, − 1.5, 800, 11, − 0.75]	(40, 100)
8	[− 200, 15, 0.3, 1800, − 10, 0]	(40, 100)
9	[− 800, − 3, 1.5, 800, 15, − 0.75]	(60, 100)
10	[− 200, − 3, 0, 1800, − 15, 0]	(60, 100)
11	[0, − 20, − 5.6, 1000, − 45, 0.2]	(80, 100)
12	[− 200, 15, 0, 1800, − 5, 0]	(80, 100)

Scenario 2: Except for the clutter rate, all other parameters are identical to scenario 1, and we assess the efficiency of the proposed parallel PHD filter in this scenario with increasing clutter rates.

Scenario 3: Except for the detection probability, this scenario is identical to scenario 1. We analyze the efficiency of the proposed parallel PHD filter in dense clutter ($\lambda$ = 300) with decreasing detection probability.

### Target tracking setup

The single target motion model^27^ adopted in the simulation is:

29 $${\varvec{x}}_{k} = ({\varvec{F}}_{k|k - 1} \otimes {\varvec{I}}_{d} ){\varvec{x}}_{k - 1} + {\varvec{w}}_{k}$$

In addition to the kinematic state, each target in the proposed FAP-PHD filter includes a feature state ${\varvec{x}}_{{f_{k} }} = [x_{{f_{d} ,k}} ,x_{{P_{SNR} ,k}} ]^{T}$. The nation $\otimes$ represents the Kronecker product, and the dimension of the identity matrix ${\varvec{I}}_{d}$ is $d$ = 2. With a covariance of $\Delta_{k|k - 1} = {\varvec{Q}}_{k|k - 1} \otimes {\varvec{I}}_{d}$ and a mean of 0, ${\varvec{w}}_{k}$ is Gaussian process noise. Here, the covariance matrix ${\varvec{Q}}_{k|k - 1}$ and the state-transition matrix ${\varvec{F}}_{k|k - 1}$ are respectively:

30 $${\varvec{Q}}_{k|k - 1} = \Sigma^{2} (1 - e\frac{{ - 2T_{s} }}{\vartheta })\left[ \begin{gathered} 0 \, 0 \, 0 \hfill \\ 0 \, 0 \, 0 \hfill \\ 0 \, 0 \, 10 \hfill \\ \end{gathered} \right]$$

31 $${\varvec{F}}_{k|k - 1} = \left[ \begin{gathered} 1 \, T_{s} \, \frac{1}{2}T_{s}^{2} \hfill \\ 0 \, 1 \, T_{s} \hfill \\ 0 \, 0 \, e^{{\frac{{ - T_{s} }}{\tau }}} \hfill \\ \end{gathered} \right]$$

with $\vartheta$ = 1 s denoting the maneuver correlation time, $\Sigma$ = 0.1 denoting the scalar acceleration standard deviation, and $T_{s}$ denoting the sampling period.

The model of single-target observation is

32 $${\varvec{z}}_{k} = \left[ \begin{gathered} r((H_{k} \otimes {\varvec{I}}_{d} ){\varvec{x}}_{k} ) \hfill \\ \theta ((H_{k} \otimes {\varvec{I}}_{d} ){\varvec{x}}_{k} ) \hfill \\ \end{gathered} \right] + {\varvec{e}}_{k}$$

where the observation matrix $H_{k} = \left[ {1 \, 0 \, 0} \right]$, $\theta ((H_{k} \otimes {\varvec{I}}_{d} ){\varvec{x}}_{k} )$ and $r((H_{k} \otimes {\varvec{I}}_{d} ){\varvec{x}}_{k} )$ are the bearing and range of state ${\varvec{x}}_{k}$, and ${\varvec{e}}_{k}$ is the observation noise. The observation noise ${\varvec{e}}_{k}$ follows Gaussian distribution with a covariance of ${\varvec{R}} = {\text{diag(}}\left[ {10,1} \right])$ and a mean of 0. The Doppler frequency of each target in the proposed FAP-PHD filter is calculated by

33 $$f_{d} = \frac{{2f_{r} v_{r} }}{c}$$

with $f_{d}$ and $f_{r}$ denoting the target's Doppler frequency and the radar frequency, $c$ denoting the speed of light, and $v_{r}$ denoting the target radial velocity. The SNR of each target at adjacent moments follows a normal distribution with mean 6 and variance 2. For each scenario, the same target trajectory is used in 100 Monte Carlo trials to record the average performance, and each trial is independent of the other. Additionally, the efficiency of four filters is assessed using computational time and optimal subpattern assignment metric (OSPA) errors^28^.

### Analysis of the results

#### Results analysis for scenario 1

**Figure 4:**
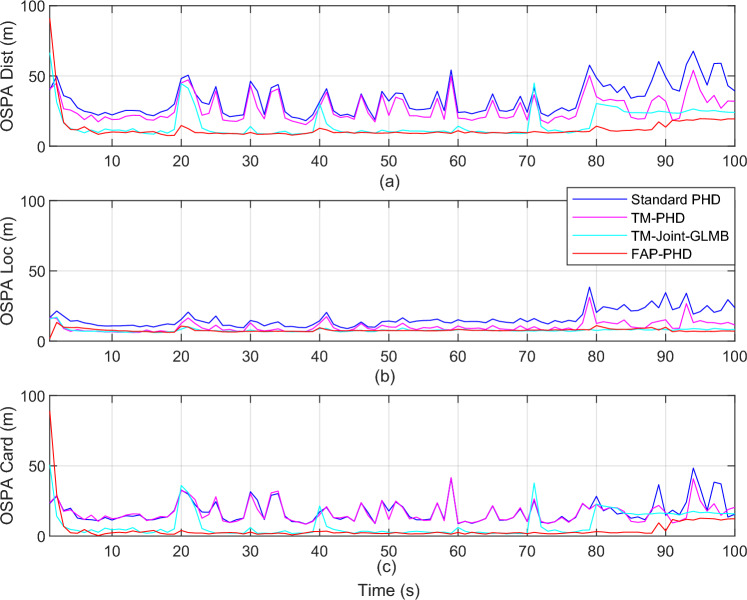
OSPA distance and its location and cardinality components for different filters ($\lambda$ = 50, $P_{D,k}$ = 0.98).

**Figure 5:**
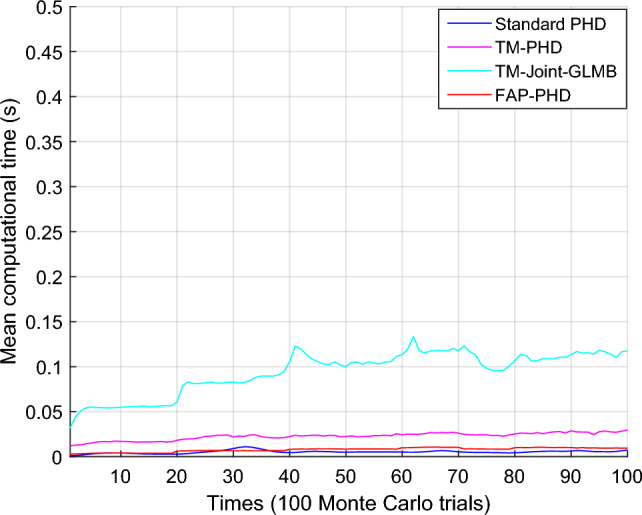
Mean computational time of different filters ($\lambda$ = 50, $P_{D,k}$ = 0.98).

Figure 4 presents the OSPA distance and its location and cardinality components for different filters. In this figure, “OSPA Dist,” “OSPA Loc,” and “OSPA Card” refer to OSPA distance, location components of OSPA, and cardinality components of OSPA, respectively. Figure 5 presents the computational time of different filters. It demonstrates that the FAP-PHD performs slightly better in location estimation and cardinality estimation than the TM-Joint-GLMB filter and outperforms the TM-PHD filter and the standard PHD filter, especially when targets disappear at $k$ = 70 s and new targets are discovered at $k$ = 20 s, $k$ = 40 s, $k$ = 60 s, and $k$ = 80 s. However, the TM-Joint-GLMB filter is more computationally expensive, and the FAP-PHD filter performs marginally higher computation time than the standard PHD filter.

When the clutter density is comparatively low, and the detection probability is relatively high, that is, $\lambda$ = 50 and $P_{D,k}$ = 0.98, the OSPA error and computing time for different filters are shown in Table 2. When compared to the standard PHD filter, the proposed FAP-PHD filter reduces the average OSPA error by 66.88%, while the average OSPA errors of the TM-PHD and TM-Joint-GLMB filters are reduced by 19.23% and 51.42%, respectively. The average computational time of the FAP-PHD filter is increased by 1.4 times, while the average computational time of the TM-Joint-GLMB filter and the TM-PHD filter is increased by 18 times and 4.3 times, respectively, when compared to the standard PHD filter. As a result, the FAP-PHD filter enhances tracking performance compared to the TM-Joint-GLMB filter without significantly increasing time cost.

**Table 2:** OSPA error and computing time for different filters ($\lambda$ = 50, $P_{D,k}$ = 0.98).

Filter	Standard PHD	TM-PHD	TM-Joint-GLMB	FAP-PHD
OSPA error (m)	33.3810	26.9609	16.2156	**11.0570**
Computing time (s)	**0.0053**	0.0227	0.0943	0.0076

Significant values are in bold.

#### Results analysis for scenario 2

**Figure 6:**
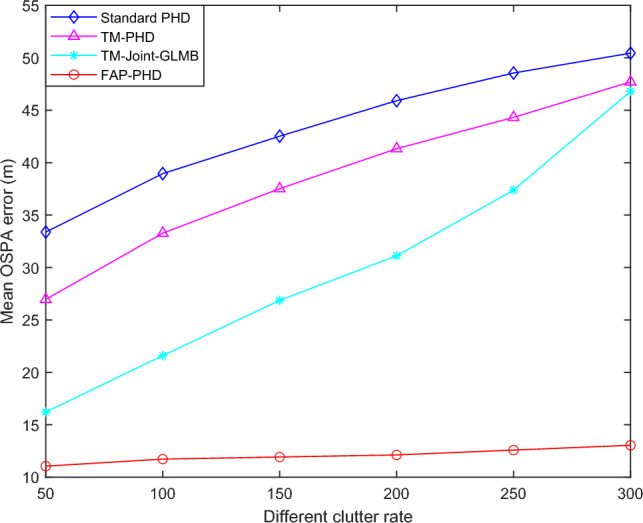
The mean OSPA performance improvement versus clutter rates.

Figure 6 shows the mean OSPA performance improvement versus clutter rates. Table 3 shows the improvement in real-time performance versus clutter rates. As can be observed in Fig. 6, the average OSPA error of the proposed FAP-PHD filter remains essentially the same when the clutter density rises, but the mean OSPA errors of the other three filters increase. The time cost of the FAP-PHD filter is less than that of the other three filters, except that it is marginally higher than the standard PHD filter at $\lambda$ = 50.

**Table 3:** The improvement in real-time performance versus clutter rates ($P_{D,k}$ = 0.98).

Filter	$\lambda$ = 50	$\lambda$ = 100	$\lambda$ = 150	$\lambda$ = 200	$\lambda$ = 250	$\lambda$ = 300
Standard PHD	**0.0053**	0.0091	0.0128	0.0169	0.0192	0.0196
TM-PHD	0.0227	0.0411	0.0544	0.0827	0.1053	0.1173
TM-Joint-GLMB	0.0943	0.1039	0.1078	0.1214	0.1319	0.1355
FAP-PHD	0.0076	**0.0077**	**0.0077**	**0.00864**	**0.0089**	**0.0097**

Significant values are in bold.

When the clutter density is relatively high, that is, $\lambda$ = 300, the proposed FAP-PHD filter reduces the average OSPA error by 74.14% compared with the standard PHD filter. In comparison, the average OSPA errors of the TM-Joint-GLMB filter and the TM-PHD filter are reduced by 7.22% and 5.43%, respectively. The time cost of the FAP-PHD filter is reduced by 50.5%, while that of the TM-PHD and the TM-Joint-GLMB filters is increased by six times and 6.9 times, respectively. The results show that in this scenario, compared with the standard PHD filter, the tracking performance of the FAP-PHD filter is improved by 74.14%, and the real-time performance is enhanced by 50.5%.

#### Results analysis for scenario 3

**Figure 7:**
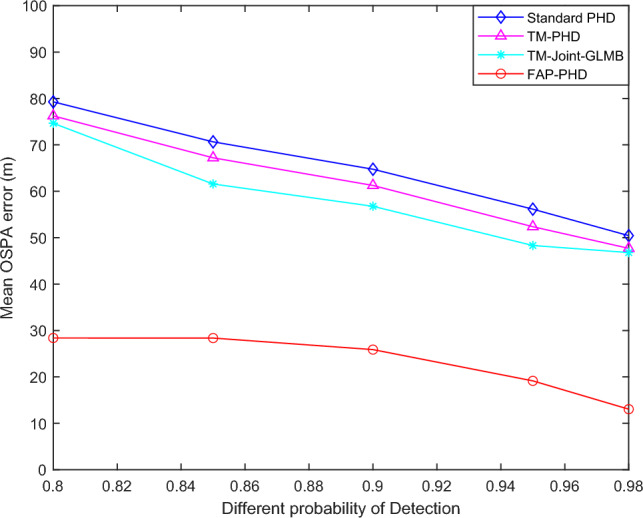
The mean OSPA performance improvement versus detection probability.

Figure 7 shows the mean OSPA performance improvement versus detection probability. Table 4 shows the improvement in real-time performance versus detection probability. The results indicated that the average OSPA errors of all four filters increase with the decrease of detection probability. However, the proposed FAP-PHD filter obviously outperforms the TM-Joint-GLMB filter, the TM-PHD filter, and the standard PHD filter. Meanwhile, the average computational time of the FAP-PHD filter is lower than that of the other three filters.

**Table 4:** The improvement in real-time performance versus detection probability.

Filter	$P_{D,k}$ = 0.8	$P_{D,k}$ = 0.85	$P_{D,k}$ = 0.9	$P_{D,k}$ = 0.95	$P_{D,k}$ = 0.98
Standard PHD	0.0263	0.0253	0.0231	0.0216	0.0196
TM-PHD	0.1394	0.1314	0.1267	0.1234	0.1173
TM-Joint-GLMB	0.1519	0.1494	0.1416	0.1398	0.1355
FAP-PHD	0.0118	0.0114	0.01057	0.0103	0.0097

When the clutter density is relatively high and the detection probability is relatively low, that is, $\lambda$ = 300 and $P_{D,k}$ = 0.8, the average OSPA error of the FAP-PHD filter is reduced by 64.19% compared with the standard PHD filter. In comparison, the average OSPA errors of the TM-Joint-GLMB and the TM-PHD filters are reduced by 5.82% and 3.81%, respectively. In the meantime, the time cost of the FAP-PHD filter is reduced by 55.1%, while that of the TM-PHD and the TM-Joint-GLMB filters is increased by 5.3 times and 5.8 times, respectively. The results show that in this scenario, compared with the standard PHD filter, the FAP-PHD filter has a tracking performance improvement of 64.19% and a real-time performance improvement of 55.1%.

All simulation results in three scenarios demonstrated that the proposed FAP-PHD filter performs exceptionally well in real-time and tracking, especially in cluttered environments. The proposed method adopts the parallel probability hypothesis density filter, which divides the measurement area into several subspaces and then takes the SNR and Doppler frequency contained in radar echoes as the features of auxiliary multi-target tracking. These features can eliminate clutter and differentiate targets from each other, thus eliminating the estimation errors introduced by sampling time diversity with less computation cost. As expected, with increasing clutter rate and decreasing detection probability, the FAP-PHD filter has a lower mean OSPA error and a faster computing time when compared to the TM-Joint-GLMB filter, the TM-PHD filter, and the standard PHD filter. Simulations are consistent with the theoretical analysis.

In summary, simulations demonstrated that the proposed method not only outperforms the TM-Joint-GLMB filter, the TM-PHD filter, and the standard PHD filter in tracking performance but also has advantages in real-time performance, especially in cluttered environments. Therefore, the FAP-PHD filter is suitable for radar application scenarios that require high real-time and tracking accuracy performance.

## Conclusion

This article proposes a radar nonlinear MTT method with a parallel PHD filter. According to the beam width of the radar antenna, the measurement area is divided into several subspaces to realize the parallel filtering on each subspace. Then, the feature information in the radar echo is used to eliminate clutter further, thus eliminating the estimation errors caused by sampling time diversity with less computation cost. Simulations demonstrated that the proposed method could address the problem of sampling time diversity in radar applications and obtain higher tracking performance with less computational cost, especially in cluttered environments. However, the proposed method still has challenges, such as extended target tracking and multi-sensor fusion. In further research, we focus on applying the proposed method to multi-sensor fusion and extended target tracking.

## Data Availability

The data generated during this study are included in this article. Matlab code is available from the corresponding author on reasonable request.
